# Ionic Liquid Agar–Alginate Beads as a Sustainable Phenol Adsorbent

**DOI:** 10.3390/polym14050984

**Published:** 2022-02-28

**Authors:** Nihal Yasir, Amir Sada Khan, Muhammad Faheem Hassan, Taleb H. Ibrahim, Mustafa I. Khamis, Paul Nancarrow

**Affiliations:** 1 Department of Chemical Engineering, College of Engineering, American University of Sharjah, Sharjah P.O. Box 26666, United Arab Emirates; nihal.yasir@gmail.com (N.Y.); aamirsada_khan@yahoo.com (A.S.K.); mhassan@aus.edu (M.F.H.); pnancarrow@aus.edu (P.N.); 2 Department of Chemistry, University of Science & Technology, Banuu 28100, Khyber Pakhtunkhwa, Pakistan; 3 Department of Biology, Chemistry and Environmental Sciences, American University of Sharjah, Sharjah P.O. Box 26666, United Arab Emirates; mkhamis@aus.edu

**Keywords:** adsorption, phenol, ionic liquids, agar, alginate, kinetic study, isotherms, wastewater

## Abstract

Cleaning wastewater containing low concentrations of phenolic compounds is a challenging task. In this work, agar–alginate beads impregnated with trihexyltetradecylphosphonium bromide ([P_66614_][Br]) ionic liquid adsorbent were synthesized as a potential adsorbent for such applications. FTIR, TGA, SEM, EDX and PZC studies were performed to characterize and understand the physicochemical properties of the adsorbent. The Fourier transformation infrared spectroscopy (FTIR) study showed that [P_66614_][Br] ionic liquid was effectively incorporated into the agar–alginate structure. TGA and SEM confirmed comparative enhanced thermal stability and porous surface, respectively. Chemical reaction rate-altering parameters, i.e., pH, contact time, initial phenol concentration and temperature, are optimized at highest phenol removal. It was found that the maximum phenol adsorption capacity and highest removal efficiency by the adsorbent occurred at pH 2, initial phenol concentration of 150 mg/L, beads dosage of 6 mg/mL and contact time of 2 h with values of 16.28 mg/g and 65.12%, respectively. The pseudo-second order model fitted the adsorption kinetics well, and the Freundlich isotherm model gave the experimental data the best fit. Analysis of thermodynamic data demonstrated that the adsorption process is fundamentally exothermic in nature, and low temperature favors spontaneity of the chemical reaction. Regeneration studies indicated that the adsorbent can at least be used for four cycles in such applications without any considerable loss in adsorption efficiency.

## 1. Introduction

Environmental pollution, with major contribution from manufacturing and extractive industries, has become one of the major challenges faced by humanity [1]. Aqueous effluents from industrial and healthcare outlets contain eco-unsafe organic and inorganic chemicals such as aromatics and heavy metals. Phenols—a type of such pollutants—are aromatic organic compounds and enter the water streams from different industries such as textile, plastic, pesticide, insecticide, paint, resin, pharmaceuticals, dyes, paper and coal extraction [2]. Phenols are noxious to living things including humans, even in very low concentrations [3].

Due to their toxic nature, aqueous steams containing phenolic wastewater (PWW) are considered as high-priority pollutants by the United States Environmental Protection Agency (US EPA) [4]. According to the World Health Organization (WHO), the allowable phenol level in water should not exceed 1 μg/L [5]. On the other hand, industrial water phenol level can reach as high as 2000 mg/L [6]. It is therefore important, from an environmental point of view, to remove phenols from wastewater before their disposal into water bodies.

In the last couple of centuries, aqueous effluents treatment, in general, and PWW treatment, in particular, have gained significant attentions from environmental stakeholders and researchers. Numerous techniques, such as solvent extraction, chemical oxidation, photocatalytic degradation and biological methods have been recognized to treat PWW [7]. However, PWW treatment at low concentrations using the abovementioned techniques is either resource-intensive, time consuming, demands delicate operations conditions or creates unwanted by-products [8]. Due to intrinsic limitations linked to these techniques, it is very important to develop alternative cheap and sustainable PWW treatment techniques [9].

Santonastaso et al. and Weidner and Ciesielczyk highlighted the significance of adsorption technique for the treatment of aqueous streams [10,11]. It is considered as a sustainable, simple, cheap and efficient technique for PWW treatment. Generally, adsorbents are readily available, broadly applicable and can be easily tailored for specific applications using surface modifiers such as ionic liquids (ILs) [12]. Their excellent properties provided grounds for the development of environmentally friendly and sustainable adsorbents. Green biopolymers such as starch, sodium alginate, chitosan, pectin and cellulose are being used as a backbone for composite hydrogel adsorbents [13]. Isawi H. synthesized nanocomposite beads from sodium alginate to treat aqueous solution containing heavy metal [14].

Surface modification often comes in handy to improve adsorption capacities at low adsorbate concentration. Since phenol is sparingly soluble in water at STP, its low concentrations can be best treated with an adsorbent surface having high chemical affinities for phenol. IL surface modified adsorbents are reported to have enhanced adsorption efficiency for PWW due to their phenol-philic surface characteristics [15,16,17]. ILs are organic salts containing organic and ionic moiety that melt below 100 °C. Due to their ease of tunability and specific thermophysical properties such as the low vapor pressure, low melting point (≥100 °C), high boiling point, conductivity, polarity and high chemical and thermal stability, ILs have become popular in academic and industrial applications [18]. Furthermore, ILs can be either hydrophilic or hydrophobic based on the selection of appropriate cations and anions. Hydrophobic ILs containing [BF_4_^−^] and [PF_6_^−^] anions are extensively used for phenol extraction from aqueous phase in liquid–liquid extraction [15]. However, these ILs contain fluorinated anions, which, under certain conditions, can produce HF upon hydrolysis in aqueous media. Recently, the modification of adsorbents with ILs has been executed to enhance their performance toward organic and inorganic contaminants. V. Archana et al. achieved a value of 9.07 mg/g for the adsorption capacity by using ionic liquid (trioctylphosphine oxide)-immobilized polymeric microcapsules to remove phenol from the aqueous medium [16].

**Figure 1:**
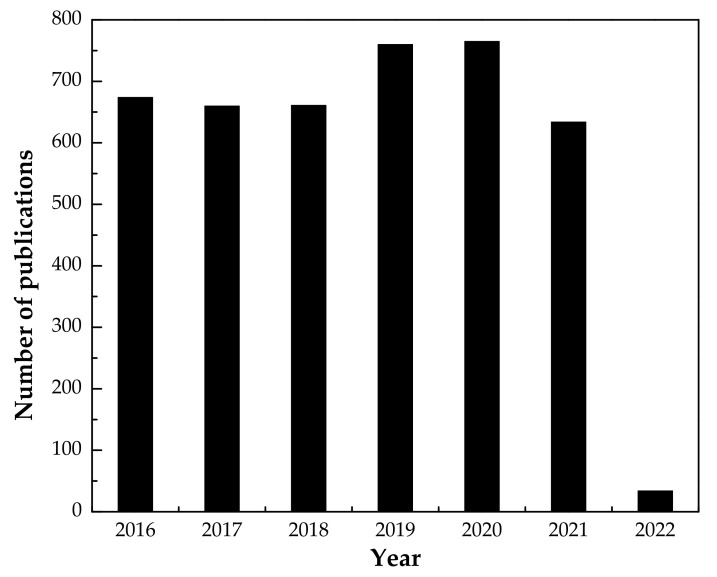
Total number of entries in web of science for phenol adsorption articles from year 2016 to 2022.

In the current research work, the IL [P_66614_][Br] was immobilized into agar–alginate biopolymers beads. The IL was immobilized by using the following procedure: (i) stabilization of agar emulsion by IL, (ii) mixing of emulsion with sodium alginate and (iii) cross-linking of the agar–alginate–IL mixture using calcium chloride. The resulting composite material, if successful, would add additional means by which low concentrations of phenols in aqueous waste streams could be removed. Known concentrations of PWW solutions were synthesized in the laboratory and were treated with the beads in batch studies to determine their adsorption potentials. A comparative study between the values of adsorption capacity, pH, temperature, removal efficiency and kinetic parameters of this work and respective values reported in the literature are given in Section 3.6. Surface, thermal and other properties of synthesized adsorbent were investigated using SEM, EDX, TGA, FTIR and PZC. Process variable such as PWW solution pH, initial concentration, adsorbent dosage, contact time and temperature were optimized to obtain the maximum phenol removal efficiency. Adsorption kinetics, isotherms and thermodynamic parameters were performed to evaluate both the adsorption capacity and its mechanism. Statistical analysis of the published articles on phenol adsorption from year 2016 to 2022 is given in Figure 1.

## 2. Experimental Study

### 2.1. Materials

All primary chemicals were of analytical grade and used as received from Sigma Aldrich, St. Louis, MO, USA unless otherwise specified. Trihexyltetradecylphosphonium bromide (C_32_H_68_BrP, ≥95%) ([P_66614_][Br]), MRS Agar (De Man, Rogosa and Sharpe) and potassium bromide (FTIR grade, ≥99%) were used. Sodium alginate having a viscosity of 2000 cP (2% Aq. solution) was purchased from SDFCL, India. Phenol (C_6_H_5_OH, 99.98%) was purchased from Fisher Scientific, Leicestershire, UK. Sodium hydroxide (NaOH, 99%), calcium chloride dihydrate, hydrochloric acid (HCl, 37%) and methanol (CH_3_OH, 99%) were all procured from Merck, Darmstadt, Germany. Distilled water was generated using an Aquatron A4000D Water Still from Stuart Equipment, Staffordshire, UK.

### 2.2. Preparation of Solutions

A solution of agar was prepared by initially heating 33 mL of distilled water to 50 °C for 15 min, then 1.65 g agar (%5 *w*/*w*) was added slowly and left to stir for 2 h and at 50 °C using a heat-stirrer (Stuart Equipment CB162, Staffordshire, UK). In this step, IL agar–alginate beads of different concentrations of [P_66614_][Br] were prepared by adding 0.5, 0.75 and 1 g of the IL to the above agar solution, left to stir for 1 h and at 70 °C and for another 1 h using a sonicator (Bandelin Sonorex, Model: RK 100 H, SN: 312.00114361.001) until a homogenous solution was obtained and no flakes could be seen. Furthermore, a 2% (*w*/*w*) sodium alginate solution was prepared by the careful addition of 0.2 g of sodium alginate to 10 mL of distilled water at 70 °C under vigorous stirring. Finally, this sodium alginate solution was added to each of the above agar–IL solutions and left to stir for 3 h to mix well and then was sonicated for another 30 min.

To prepare the phenol stock solution, a gram of phenol was dissolved in a liter of distilled water. Furthermore, working phenol solutions of different concentrations were synthesized by diluting the abovementioned parent stock solution. About 0.1 M NaOH and 0.1 M HCl were used to adjust the pH of the phenol solution. The pH was measured by a pH meter (Oakton pH 510 series, Sepang, Malaysia).

### 2.3. Preparation of IL–Agar–Alginate Beads

**Figure 2:**
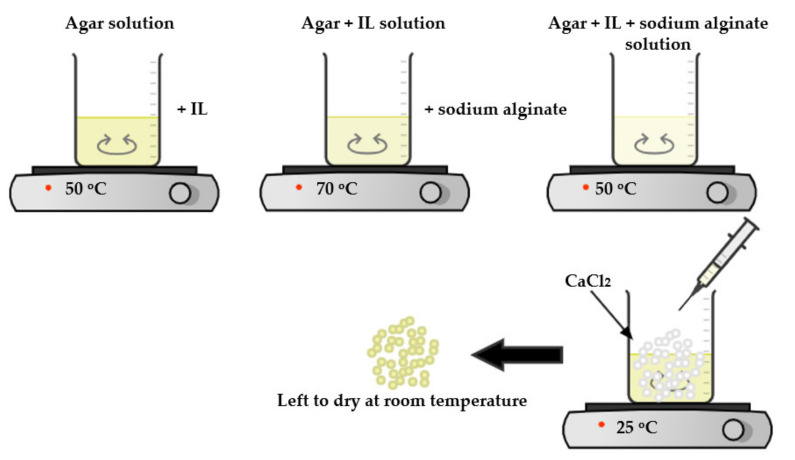
Schematic diagram for IL–agar–alginate beads preparation.

**Figure 3:**
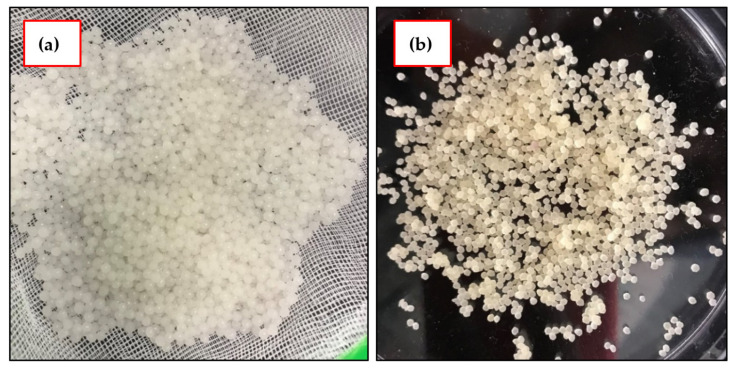
The prepared IL–agar–alginate beads (**a**) wet and (**b**) dry.

A 5% (*w*/*w*) calcium chloride solution was prepared by adding 5 g of calcium chloride to 100 g distilled water. To prepare IL–agar–alginate beads, liquid mixture (explained earlier) containing these ingredients was syringed into 5% calcium chloride aqueous solution. This process yielded uniform-sized circular adsorbent beads. To improve cross-linking and stability of the prepared adsorbent, it was left in the same solution under regular stirring (WiseStir MSH-20D, Dublin, Ireland). After 24 h, the beads were rinsed with distilled water many times until the washing solutions reached a neutral pH and then were left for air drying at 25 °C. The schematic diagram for beads preparation is shown in Figure 2. Wet beads, as can be seen, have smooth spherical shape with a diameter of about 3–4 mm. The diameter of the beads was decreased to ~1 mm after air drying, but they preserved their spherical structure Figure 3.

### 2.4. Characterization and Instruments

The FTIR (Fourier transform infrared) spectra were recorded within a range of 4000–500 cm^−1^ (Perkin Elmer: Nicolet 6700, Thermo Scientific, Waltham, MA, USA). Scanning electron microscopy (SEM) was used to observe the surface morphology of the adsorbent (TESCAN Vega 3, Warrendale, PA, USA). SEM was fitted with a detector for energy dispersive X-ray spectroscopy (SEM–EDX). The attached EDX instrument was operated at 30 kV, and samples were gold-plated prior to SEM–EDX analysis using a blazers sputtering device. Furthermore, thermal degradation analysis of the adsorbent beads was conducted using thermogravimetric analysis (TGA) (SHIMADZU DTG-60AH) from 50 to 550 °C under N_2_ atmosphere with a heating rate of 10 °C/min. In addition, a UV-vis spectrophotometer gauged the phenol solution concentration (Evolution 201, Thermo Scientific, Shanghai, China).

The pH point zero charge (pH_pzc_) for adsorbent was gauged by suspending 10 mg of beads in 10 mL of 0.01 M NaCl aqueous solution, which was agitated in an orbital shaker (Daihan Scientific Incubator, Wonju, Korea) for 24 h. The solution’s initial pH (5.8) was adjusted in the acidic region (2–6) by appending 0.1 M HCl and in the basic region (8–12) using 0.1 M NaOH solution. The resulting solutions pH was quantified with a pH meter (Oakton pH 510 series, Sepang, Malaysia), and the value of pH_pzc_ was obtained by plotting pH_f_ against pH_i_. The point of intersection was the pH_pzc_ for the IL–agar–alginate beads [19].

### 2.5. Adsorption Study

Investigation of phenol adsorption using IL–agar–alginate bead adsorbent was carried out in batch adsorption mode. In a typical experiment, a certain mass of adsorbent beads was added to 10 mL aqueous solution at a desired pH having known adsorbate concentration. The mixture was then shaken for a certain time period. The adsorbent was then isolated from the solution, and UV-vis absorbance was recorded at λ_max_ = 270 nm for phenol quantification. The adsorption capacity (*q_e_* (mg/g)) and percent removal efficiency (%*R*) were calculated using Equations (1) and (2), respectively.

(1) $$q_{e}=\left(C_{i}-C_{f}\right)\times \frac{V}{m}\;$$

(2) $$\%R=\frac{\left(C_{i}-C_{f}\right)}{C_{f}}\times 100\;$$

where $C_{i}$ and $C_{f}$ are the initial and the final concentrations (mg/L) of the phenol solution at equilibrium, respectively, V is the volume of the phenol solution in litres and m is the IL–agar–alginate beads amount in grams.

The obtained data were analyzed for the adsorption kinetics and adsorption isotherms using the models that will be described below. To define the thermodynamic parameters for the adsorption of phenol on the new synthesized IL–agar–alginate beads, the adsorption experiment was run under optimized conditions at three different temperatures: 25, 35 and 45 °C.

### 2.6. Desorption/Regeneration Study

The batch regeneration studies were performed using two different desorbing solutions, i.e., hot water and methanol. The experiment was conducted by adding 10 mg of the adsorbent to a 10 mL solution of phenol (*C_i_* = 150 mg/L) at 25 °C. After 2 h, the bead adsorbent was filtered and treated with 20 mL of the desorbing agent at 50 °C to desorb phenol from its surface. This procedure was repeated four times to study the adsorbent regeneration potential.

## 3. Results and Discussion

### 3.1. Characterization

#### 3.1.1. FTIR Analysis

**Figure 4:**
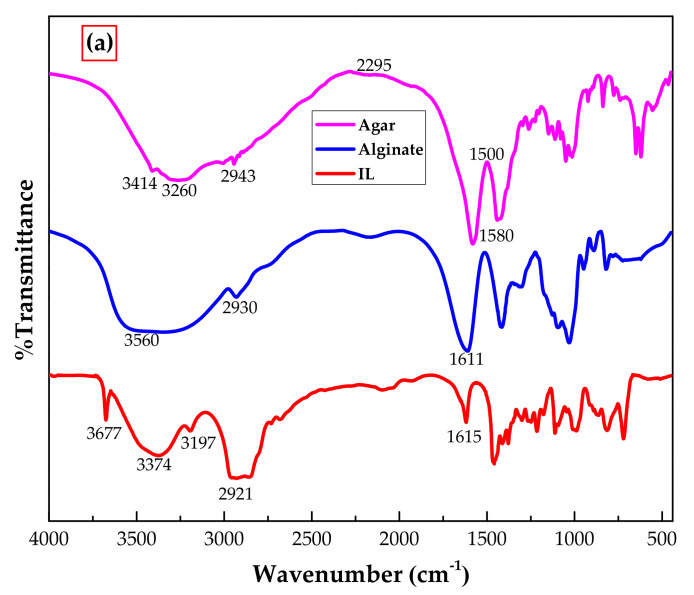

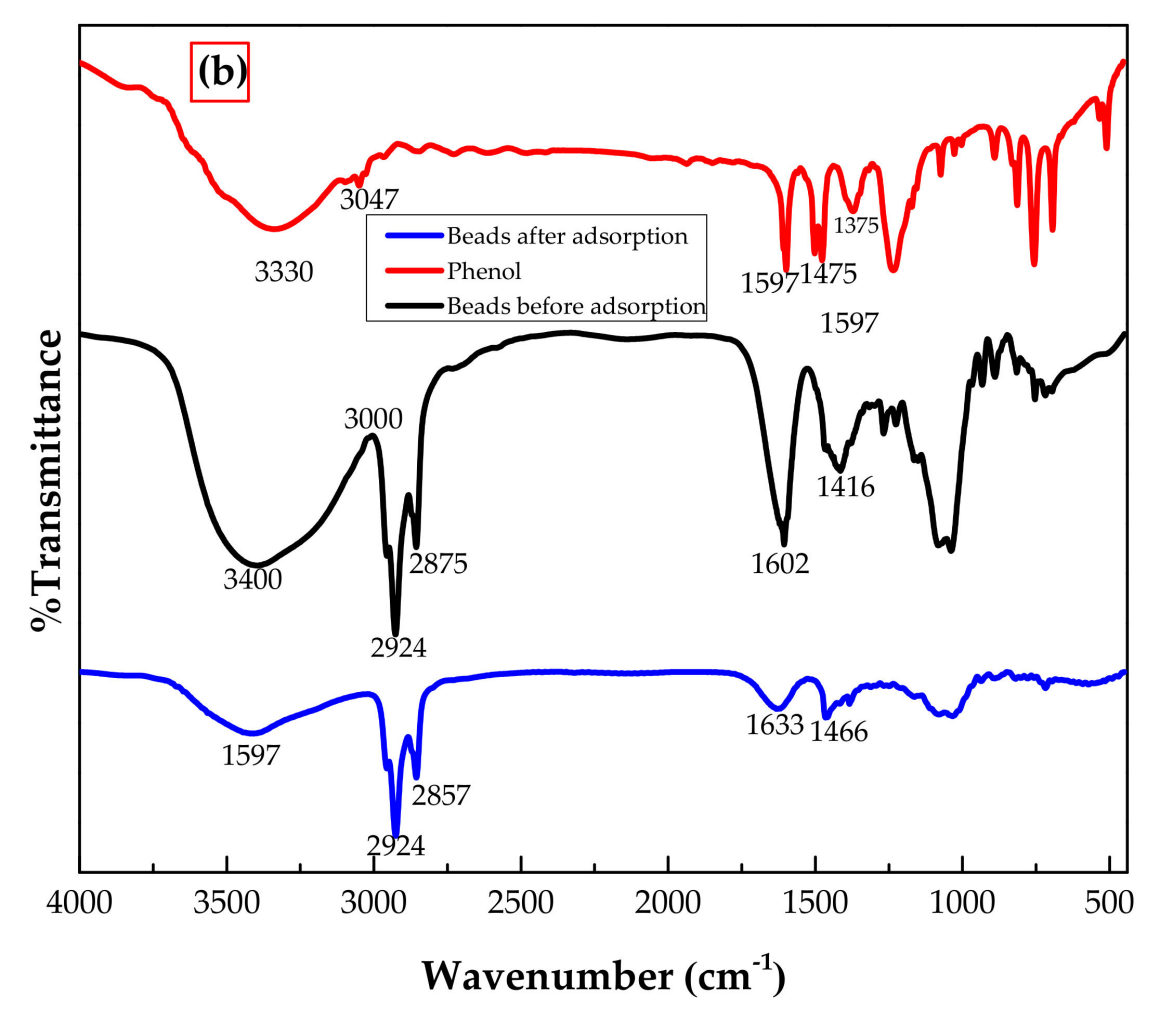
FTIR spectra of (**a**) IL, alginate and agar (**b**) Phenol and IL–agar–alginate bead before and after phenol adsorption.

Functional groups available on adsorbent, its precursors and phenol were determined using FTIR analysis. Figure 4a shows the FTIR spectrums of [P_66614_][Br], agar and sodium alginate which are further explained in detail elsewhere in literature [20,21,22]. Figure 4b shows phenol and IL–agar–alginate bead IR spectra before and after phenol adsorption. For IL (Figure 3a), C–H stretching and bending vibrations are given at 2900 and 1450 cm^−1^ peaks, respectively. The bands observed at 1200, 1255, and 1380 cm^−1^ can be assigned to -CF_3_, -CF_3_SO_3_ and -SO_3_ bond stretching [20]. The vibrations observed at 720 and 800 cm^−1^ could be due to the presence of F-P-F and P-F, respectively. [BF_4_]^−^-characteristic frequencies are given at 1000 cm^−1^ [20]. The spectrum of alginate (Figure 3a) shows alcoholic O–H and ether’s C–O–C stretching vibrations at band 3360 and 1040 cm^−1^, respectively. Moreover, C–H stretching vibrations can be seen at 2930 cm^−1^, while absorption bands shown at 1612 and 1412 cm^−1^ are linked to symmetric and asymmetric stretching vibrations of COO^−^ [22]. For agar, wide absorption bands around 3400–3000 cm^−1^ highlight available O–H groups on agar surface. Absorption stretching at 2943 cm^−1^ is due to C–H of methoxyl group, while stretching vibrations of CO and NH groups of conjugated peptide bonds are given at 1580 cm^−1^ [21].

The peaks present in the IL, alginate and agar at 2900, 2930 and 2943 cm^−1^, respectively, all shifted to 2924 cm^−1^ in the IL–agar–alginate bead spectrum. It is worth mentioning that the FTIR spectrum of the IL–agar–alginate bead contains all the characteristic peaks present in its constituent chemicals, i.e., IL, agar and alginate. Figure 4b shows FTIR spectra of adsorbent after phenol adsorption is substantially modified. This highlights successful phenol adsorption. Moreover, the following peaks shifts were observed upon phenol adsorption: 1633 to 1602 cm^−1^, 1466 to 1416 cm^−1^, 1073 to 1033 cm^−1^ and 718 cm^−1^ to 689 cm^−1^. All these changes could be attributed to successful interaction of phenol with the adsorbent [23].

#### 3.1.2. Thermogravimetric Analysis

**Figure 5:**
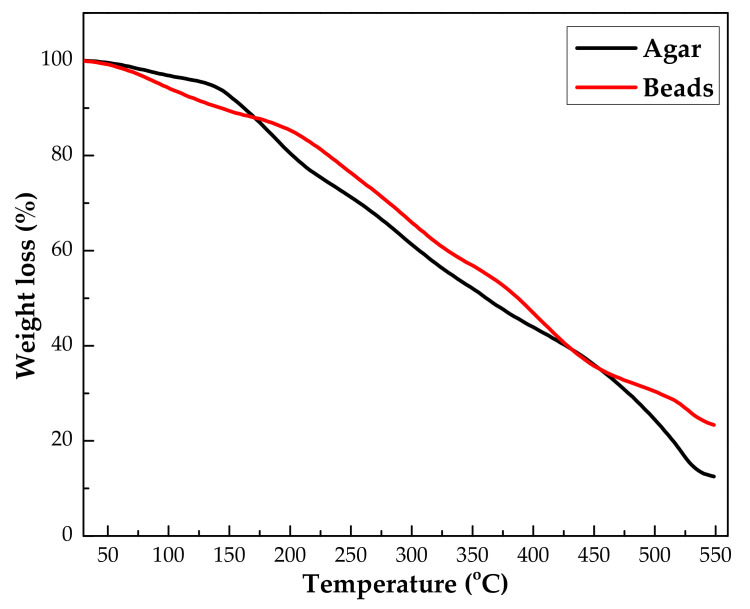
TGA curve for agar and agar–alginate-IL beads.

The thermal stability of both agar powder and IL–agar–alginate beads were tested by thermogravimetric analysis in a temperature range of 50–550 °C with a heating rate of about 10 °C/min, under a dynamic nitrogen atmosphere. Thermal analysis illustrates change in weight loss with increasing temperature, as shown in Figure 5. It shows that the thermal degradation of agar and IL–agar–alginate beads took place in three distinct stages. The first stage occurred in range 50–120 °C and is attributed to moisture reduction. The second step occurred in the range of 200–400 °C and is attributed to the active pyrolysis with drastic weight loss. The third step occurred in the range of 400–500 °C and is attributed to decomposition of the organic moieties in the compounds. An additional weight loss was observed very slowly up to 550 °C. A similar pattern was found for alginate beads in the thermal degradation literature [24].

#### 3.1.3. SEM–EDX Analysis

**Figure 6:**
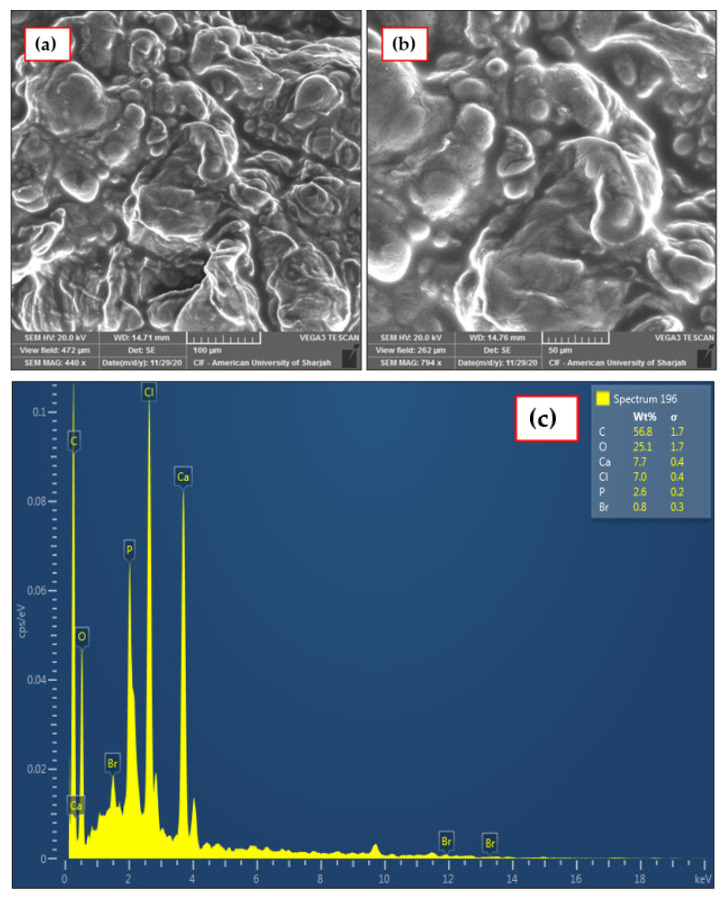
SEM images for adsorbent bead at two different magnifications (**a**) 100 µm and (**b**) 50 µm. (**c**) EDX analysis of adsorbent bead.

SEM–EDX was employed to analyze the surface morphology and the texture of the IL–agar–alginate beads. The images taken for the sample under various magnifications are shown in Figure 6a,b. The SEM images showed that the beads have a porous–rough surface with several grooves. This rough surface contains different sizes of pores, which are good for adsorption applications [25,26]. Therefore, these pores provide a way to accumulate phenol molecules. A previous study showed that pores and cavities in alginate beads were responsible for better phenol adsorption [27]. The elemental investigation of the beads was determined using EDX analysis and is shown in Figure 6c.

The presence of the phosphate in adsorbent confirms successful encapsulation of phosphonium-based IL in the agar–alginate composite. Table 1 summarizes the elemental analysis of different elements present in the adsorbent.

#### 3.1.4. The pH of Point Zero Charge Analysis (pH_pzc_)

**Figure 7:**
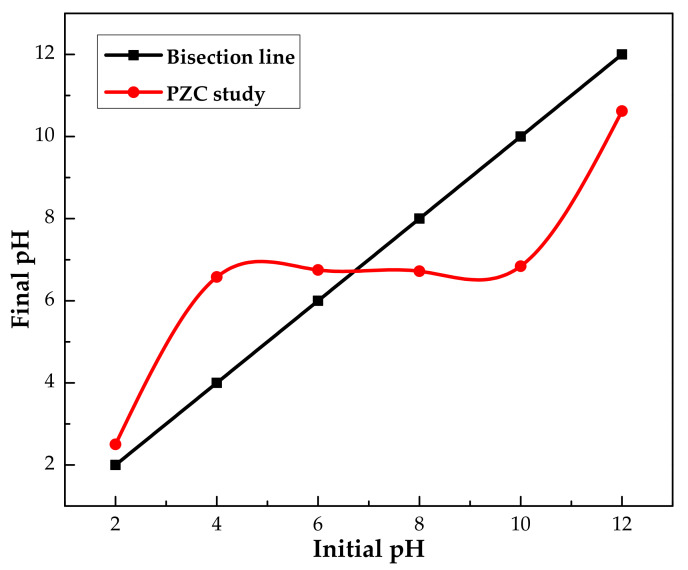
pH_pzc_ analysis of agar–alginate-IL beads study at room temperature.

The point of zero charge (pH_pzc_) is generally described as the pH at which the charge of the adsorbent (i.e., the surface of the IL–agar–alginate bead) is equivalent to zero. It is introduced in colloidal flocculation studies to explain how pH affects adsorption [28]. pH_pzc_ curves for IL–agar–alginate bead are presented in Figure 7. Inspection of this figure reveals that the point of zero charge is 6.7 for our adsorbent. Therefore, the bead has a positive charge on its surface at pH < pH_pzc_ and, subsequently, a negative charge at pH > pH_pzc_ [29,30]. Maximum adsorption of phenol was observed at pH 2, which is well supported by the point zero charge study.

### 3.2. Phenol Adsorption Study

#### 3.2.1. Effect of IL/Agar Ratio and Adsorbent Dosage

The initial findings have shown that phenol adsorption increased by impregnating agar–alginate beads with IL. Phenol removal increased from 4.00 to 50.9 mg/g with the addition of 0.014 g/g (IL/agar–alginate) IL in agar–alginate solution (IL/agar–alginate). However, further additions of IL, i.e., 0.021 or 0.028 g/g (IL/agar–alginate) to agar–alginate solution, leads to a reduction in adsorption performance. This decreasing trend may be due to the change in the morphology of the solid surface that reduced the active sites for phenol adsorption. Therefore, an overall amount of 0.5 g was selected, for further studies, as the optimized quantity of the [P_66614_][Br] per 35.7 g of agar–alginate solution in the preparation of adsorbent.

**Figure 8:**
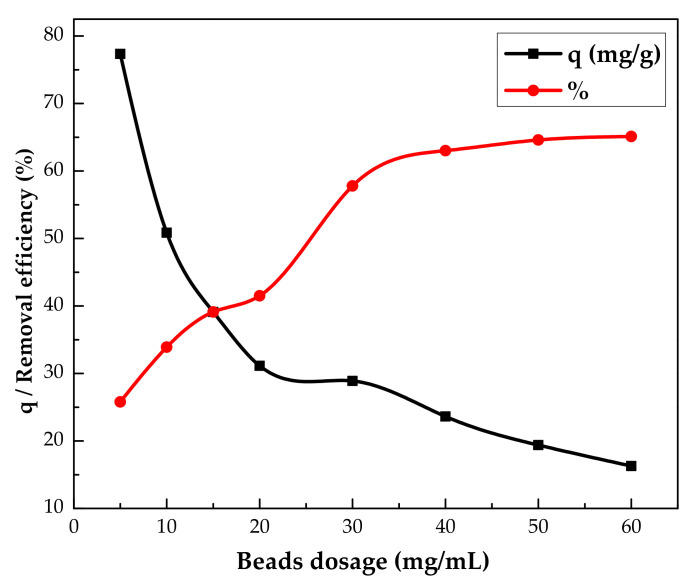
Effect of IL–agar–alginate beads dosage on the adsorption capacity and on the removal efficiency of phenol (*Ci* = 150 mg/L, pH of 2, 150 rpm, 2 h and at 25 °C).

The effect of the IL–agar–alginate bead dosage was assessed on the phenol percent removal efficiency (%*R*) and adsorption capacity (*q*). Adsorbent dosage varied in the range of 5.0–60.0 mg/100 mL, and results are presented Figure 8. The figure shows the efficiency of phenol adsorption increases sharply from 25.8 to 63.0% by increasing the adsorbent dosage from 5.0 to 40 mg/100 mL. This observation could be attributed to the existence of larger active sites for adsorption. The percent removal efficiency of adsorption was almost constant at 65% beyond 40 mg/100 mL. However, the adsorption capacity was observed to decrease from 77.4 to 16.3 mg/g when the adsorbent dosage was increased from 5.0 to 60.0 mg/100 mL. This decrease in the adsorption capacity might be credited to the inverse relation between the amount adsorbed and the mass of the adsorbent. The rise in the efficiency of removal and the fall in the adsorption capacity with the adsorbent amount is very general and has already been reported elsewhere in the literature [31]. Therefore, an optimum adsorbent dosage of 4.0 mg/mL was selected and used in further studies.

#### 3.2.2. Effect of pH

**Figure 9:**
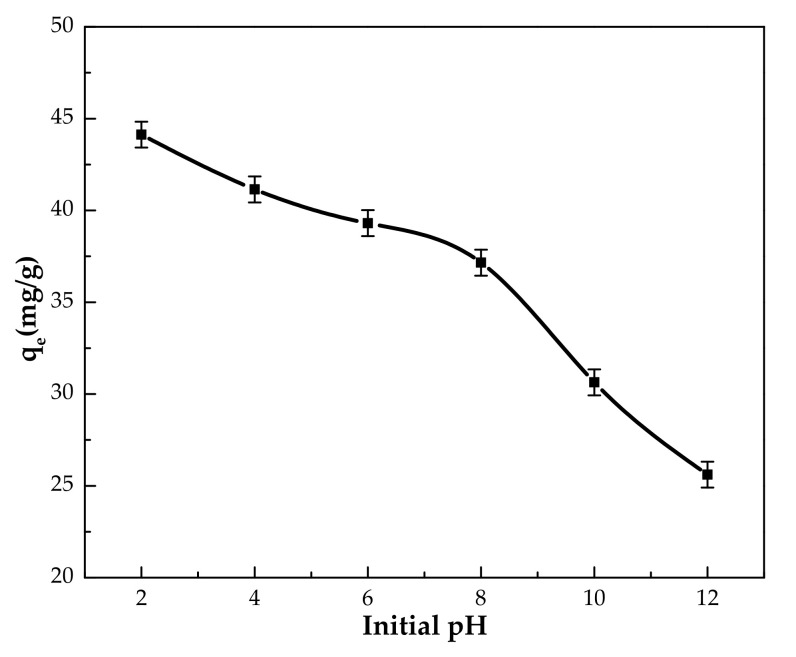
The effect of initial pH of phenol solution on the adsorption capacity by IL–agar–alginate beads (*Ci* = 100 mg/L, 150 rpm, 24 h, beads dosage = 1 mg/mL and at 25 °C).

The solution pH is an important parameter which often affects the interaction between the adsorbent and adsorbate during the adsorption process [32]. It may affect the capacity of adsorption (*q*) by altering the surface charge of adsorbents and phenol species [33]. Therefore, the impact of pH of the initial phenol solution on the adsorption capacity (*q_e_*) was investigated in the range of 2–12 and is shown in Figure 9. The figure indicates that the adsorption capacity was the highest at pH 2 with a value of 44.1 mg/g. It is known that phenol exists in its molecular form at low pH and ionizes to phenolate ions at high pH [27]. Considering this information and the pH_pzc_, the pH dependence of the adsorption capacity could be attributed to the ion–dipole interaction between the positive charge on the surface at low pH and partial negative charge on O atom in phenol. The decrease in the adsorption capacity at higher pH could be attributed to the electrostatic repulsion between the negatively charged surface at pH > 6.8 (as revealed from pH_pzc_ results) and the phenolate ions. Therefore, a pH of 2 was adopted as the optimum condition for further experiments.

#### 3.2.3. Effect of Contact Time

**Figure 10:**
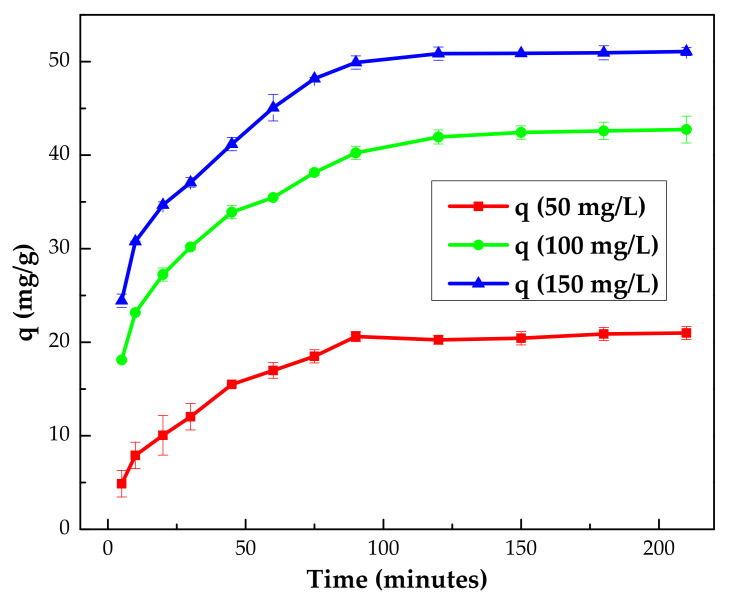
Effect of time on phenol adsorption onto the IL–agar–alginate beads (*Ci* = 150 mg/L, pH of 2, 150 rpm, 2 h, beads dosage = 1 mg/mL and at 25 °C).

The kinetics of phenol adsorption on IL–agar–alginate beads were examined at three initial concentrations, namely 50, 100 and 150 mg/L, over contact times ranging from 5 to 210 min at an adsorbent dosage of 1 mg/mL, pH 2 and 25 °C. The results are presented in Figure 10. Within the first 80 min, all the adsorption curves rose rapidly and then leveled off. The fast adsorption of phenol at lower contact time is attributed to a wide range of functional groups available for phenol adsorption. However, with the passage of time, these functional groups become occupied by the phenol molecules, which slows down the adsorption of phenol. During the adsorption process, the adsorption rate gradually decreases until equilibrium is attained. The reduction in the rate of phenol adsorption may also be due to the gradual dispersal of both ions through the bulk of the adsorbent. This higher adsorption of pollutants during the initial period of contact time and lower adsorption rate at higher contact time is typical for batch adsorption and is reported elsewhere in literature [34]. The results demonstrate that the optimum contact time for the removal of phenol by the synthesized beads is 120 min.

#### 3.2.4. Effect of Initial Concentration

**Figure 11:**
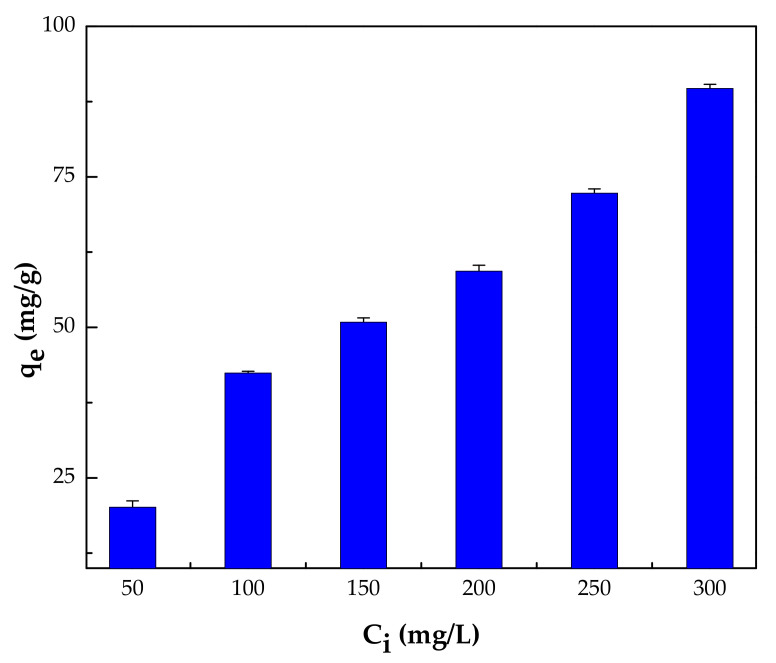
Effect of initial phenol concentration on the adsorption capacity (pH of 2, 150 rpm, 2 h, beads dosage = 1 mg/mL and at 25 °C).

To analyze the influence of the initial concentration of the phenol solution on the adsorption efficiency, the phenol concentration was ranged from 50 to 300 mg/L using 10 mL of phenol solution of 2 pH and adsorbent dose of 1 mg/mL at room temperature. The results are displayed in Figure 11. The findings illustrate that the adsorption capacity for phenol increased significantly from 20.1 to 89.7 mg/g when the initial phenol concentration was raised from 50.0 to 300.0 mg/L. The increase in the adsorption capacity as the initial phenol concentration increases is due to the transfer of more phenol molecules to the surface of IL–agar–alginate beads from the bulk. This rise in the adsorption capacity may be attributable to a higher driving force for the mass transfer of phenol from solution to the active sites available on the adsorbent surface [35].

#### 3.2.5. Effect of Temperature

**Figure 12:**
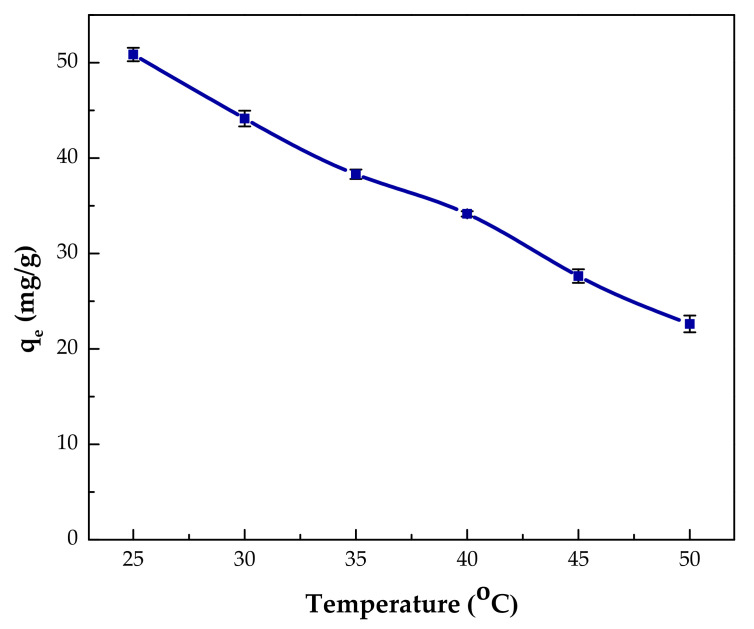
Effect of temperature on phenol adsorption capacity of IL–agar–alginate beads (*Ci* = 150 mg/L, pH of 2, 150 rpm, 2 h and beads dosage = 1 mg/mL).

Temperature is one of the major factors that influences the adsorption capacity; hence, it is necessary to investigate its effect. The temperature affect was studied in the range of 25 to 50 °C. Meanwhile, other parameters were kept at their already optimized values, for example, initial concentration of phenol at 150 mg/L; adsorbent dosage at 1 mg/mL; pH 2; and contact time at 120 min. Figure 12 displays change of adsorption capacity with temperature varition. It is clear that the adsorption capacity decreased from 50.9 to 22.6 mg/g when the temperature was raised from 25 to 50 °C, indicating the exothermic nature of adsorption process [3,4,36]. A possible explanation for this behavior is that negative enthalpy of the adsorption process encourages formation of intermolecular forces between the adsorbent and the adsorbate [19].

### 3.3. Adsorption Models

#### 3.3.1. Adsorption Kinetics

**Figure 13:**
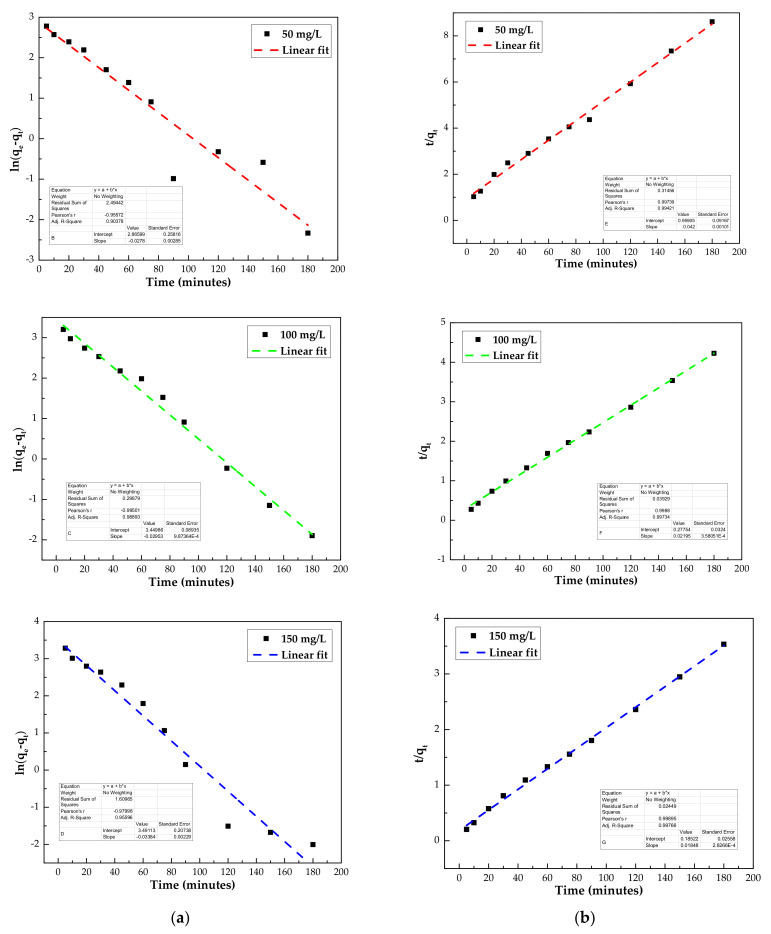
Kinetics plots for adsorption of phenol by IL–agar–alginate beads at 25.0 °C (using pseudo-first order model (PFO) (**a**) and pseudo-second order model (PSO) (**b**)) at different initial concentrations of phenol.

To investigate the kinetics of phenol adsorption on the IL–agar–alginate beads, two models, pseudo-first order (PFO) and pseudo-second order (PSO), were used Figure 13 to fit the experimental data, as shown in Equations (3) and (4), respectively [37].

(3) $$ln\left(q_{e}-q_{t}\right)=lnq_{e}-k_{1}t$$

(4) $$\frac{t}{q_{t}}=\frac{1}{k_{2}q_{e}^{2}}+\frac{1}{q_{e}}t$$

where *k*_1_ (min^−1^) and *k*_2_ (g mg^−1^ min^−1^) are the rate constants of both pseudo-first order and pseudo-second order models, respectively. Calculated kinetic parameters are listed in Table 2. Kinetic parameters for the adsorption of phenol onto the IL-agar-alginate beads at 25 °C. Regression coefficient value of pseudo-second order model (*R*^2^ > 0.99) illustrates that the adsorption of phenol follows this behavior. Experimental adsorption capacity 65 mg/g has a close agreement with theoretical *q_e_* obtained from the pseudo-second order model. These findings confirm applicability of pseudo second order mechanism for the adsorption process.

#### 3.3.2. Adsorption Isotherms

**Figure 14:**
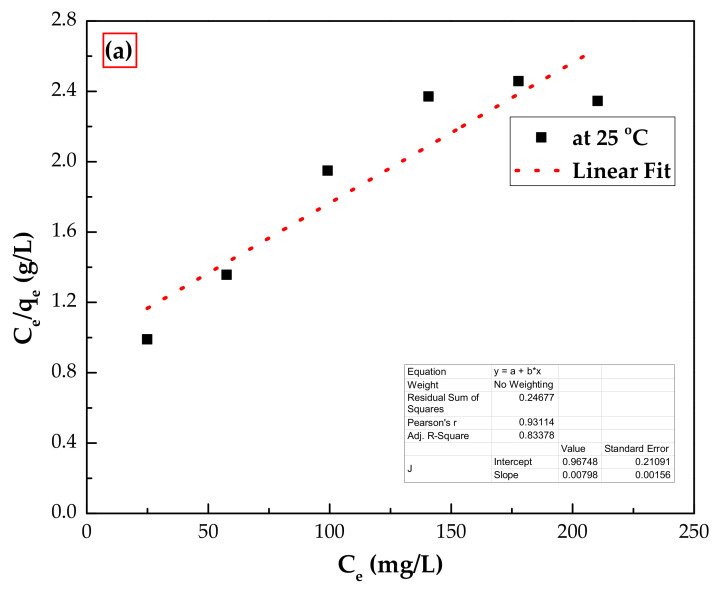

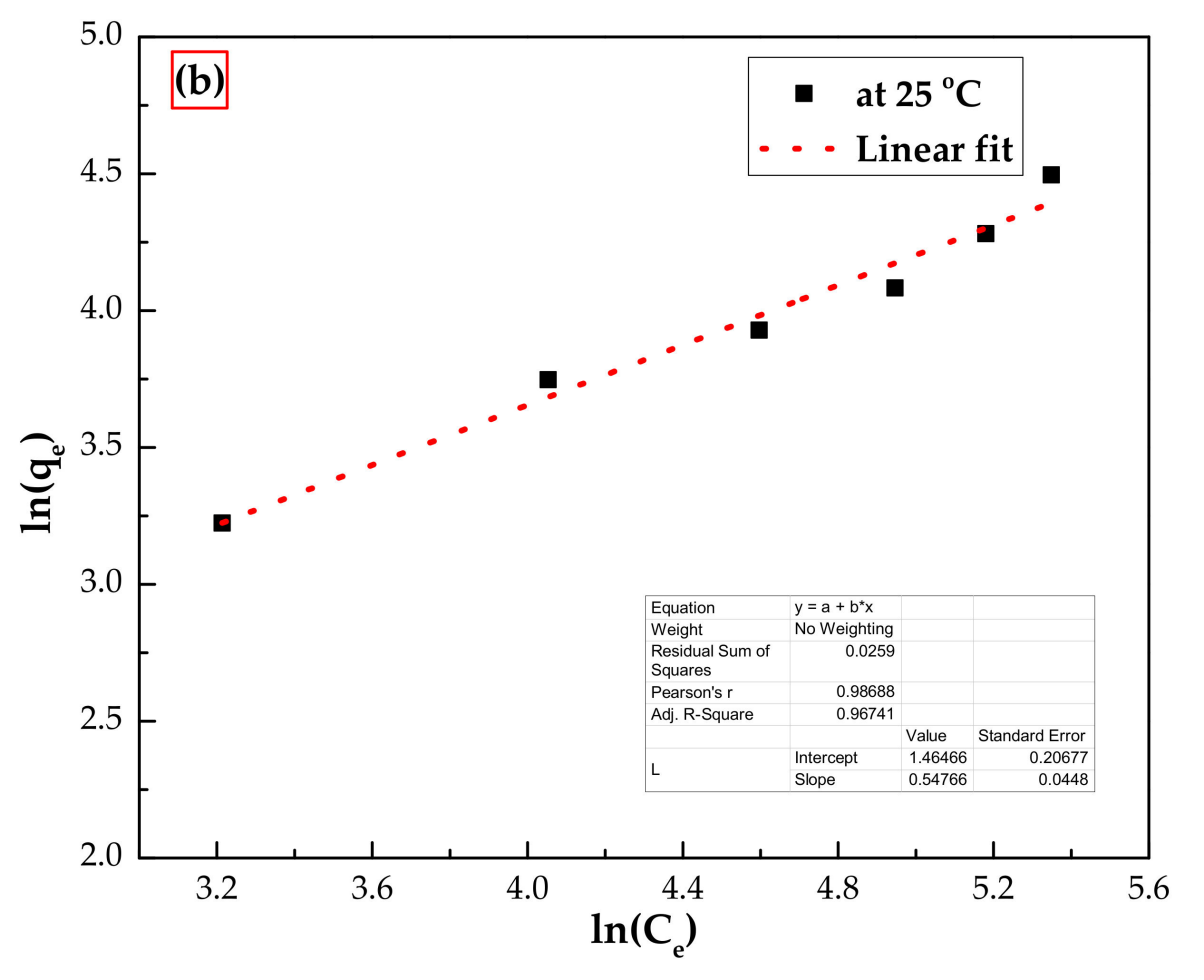
Adsorption isotherm plots for phenol adsorption on IL–agar–alginate beads using (**a**) Langmuir and (**b**) Freundlich models at 25 °C.

Phenol adsorption isotherms were tested at room temperature. In this case, two well-known isothermal models were used to simulate the isothermal experimental data, namely, Langmuir and Freundlich models [34]. The linear forms of these models are given in Equations (5) and (6), respectively.

(5) $$\frac{C_{e}}{q_{e}}=\frac{1}{bq_{max}}+\frac{C_{e}}{q_{max}}\;$$

(6) $$lnq_{e}=lnk_{f}+\frac{1}{n}lnC_{e}\;$$

where *C_e_* (mg/L) is the concentration of phenol remaining in the aqueous phase at equilibrium, *q_m_* is the maximum adsorption capacity of phenol by IL–agar–alginate beads (mg/g), *b* is the Langmuir adsorption constant (L mg^−^^1^), related to the adsorption free energy, *k_f_* (L mg^−^^1^) and n is the Freundlich constant. The linear plots of both isotherms are displayed in Figure 14. All calculated isothermal parameters and correlation coefficients are shown in Table 3. As can be seen, the phenol adsorption experimental data on the IL–agar–alginate beads is well described by the Freundlich isotherm model with *R*^2^ = 0.956 as compared to 0.835 for the Langmuir isotherm model.

### 3.4. Thermodynamic Analysis

To determine the thermodynamic parameters for phenol adsorption onto the IL–agar–alginate beads from the aqueous phase, the adsorption experiments were performed over the temperature range from 25 to 45 °C. The equation which was used to determine the change in Gibbs free energy (Δ*G*°, kJ mol^−^^1^) is given in Equation (7).

(7) $$\Delta G^{{}^{\circ}}=-RTlnK^{{}^{\circ}}$$

**Figure 15:**
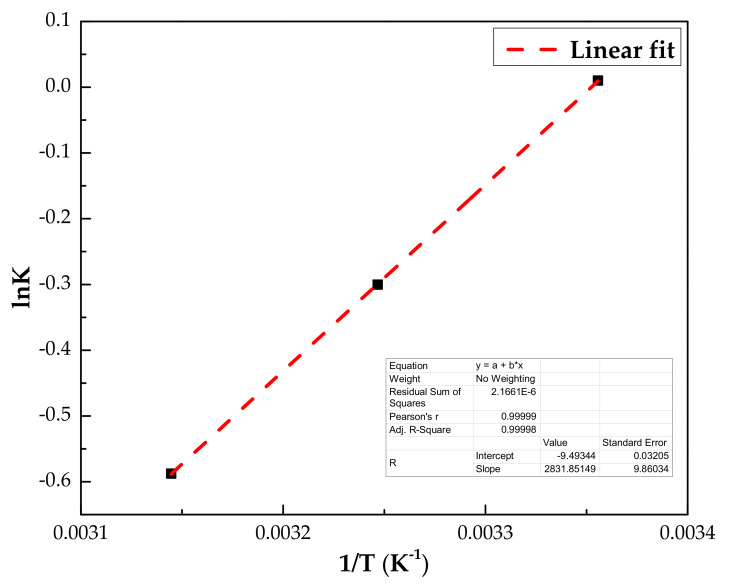
Van’t Hoff plot for the adsorption of phenol on the IL–agar–alginate beads.

The other thermodynamic parameters such as the standard enthalpy change (Δ*H*°, kJ mol^−^^1^) and standard entropy change (Δ*S*°, kJ mol^−^^1^K^−^^1^) for phenol adsorption were calculated via the van’t Hoff plot (*lnK*° against 1/*T*), assuming constant Δ*H*° as given in Equation (8). Equation (9) represents the mathematical formula for determining the equilibrium constant [38]:

(8) $$lnK^{{}^{\circ}}=\frac{-\Delta H^{o}}{R}\frac{1}{T}+\frac{\Delta S^{{}^{\circ}}}{R}\;$$

(9) $$lnK^{{}^{\circ}}=ln\left(\frac{q}{C_{f}}\right)$$

where *K°* is the equilibrium constant obtained from the ratio of *q* and *C_f_* when adsorption experiments were run at various temperature, *R* is the universal gas constant (8.314 J/mol K) and *T* is the temperature (*K*). Figure 15 displays the Van’t Hoff plot, which gives Δ*H*° and Δ*S*° from the slope and intercept, respectively. The values of the thermodynamic parameters obtained for phenol adsorption on IL–agar–alginate beads are depicted in Table 4. It has been noted that the consistent increase in the value of Δ*G*° with an increase in temperature of the adsorption process suggests that adsorption is only spontaneous and feasible at lower temperatures. The negative Δ*S*° value shows a decrease in randomness throughout the process in the solid–liquid interface. Moreover, the negative Δ*H*° value indicates the exothermic nature of the adsorption process.

### 3.5. Recyclability and Reusability

**Figure 16:**
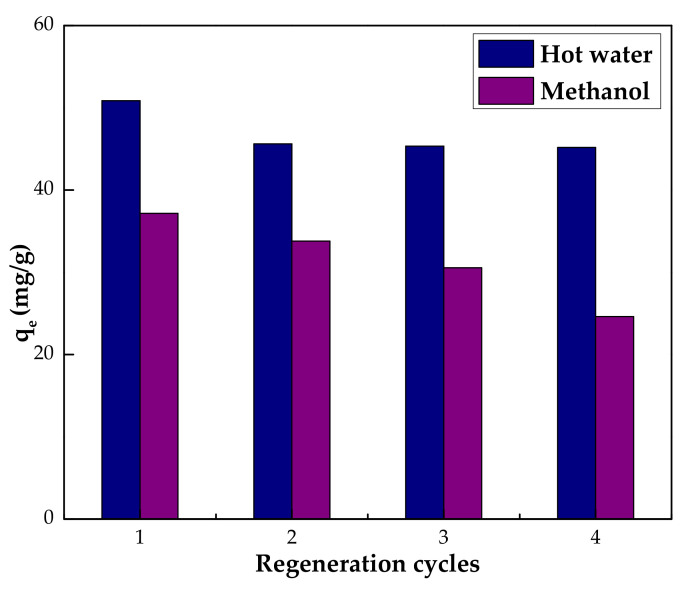
Regeneration and reusability of IL–agar–alginate beads.

The recycling of an adsorbent after adsorption is essential for it to be considered economically viable for industrial applications. The process of desorption/regeneration was performed using methanol and hot water (50 °C). The IL–agar–alginate beads were recycled and reused four times, and the results are shown in Figure 16. Hot water proved to be more than methanol in the regeneration study. It was found that adsorption efficiency slightly decreased when using hot water in the second cycle but remained constant in the subsequent cycles. The adsorption efficiency of the IL–agar–alginate beads was 33.9, 30.41, 30.25 and 30.12% for the first, second, third and fourth cycles, respectively, which implies that the IL–agar–alginate beads composite has potential as a renewable adsorbent. Values for the adsorption capacity and removal efficiency when using methanol are listed in Table 5 below.

### 3.6. Comparative Study with Literature Values

The maximum adsorption capacity of the IL–agar–alginate beads for phenol removal was compared with that of other adsorbents already reported in the literature. The comparison given in Table 6 and Table 7 confirms that the synthesized IL–agar–alginate beads as a higher adsorption capacity than similar reported adsorbents. This proved that the IL could improve the efficiency of agar–alginate beads for phenol removal.

### 3.7. Adsorption Mechanism

**Figure 17:**
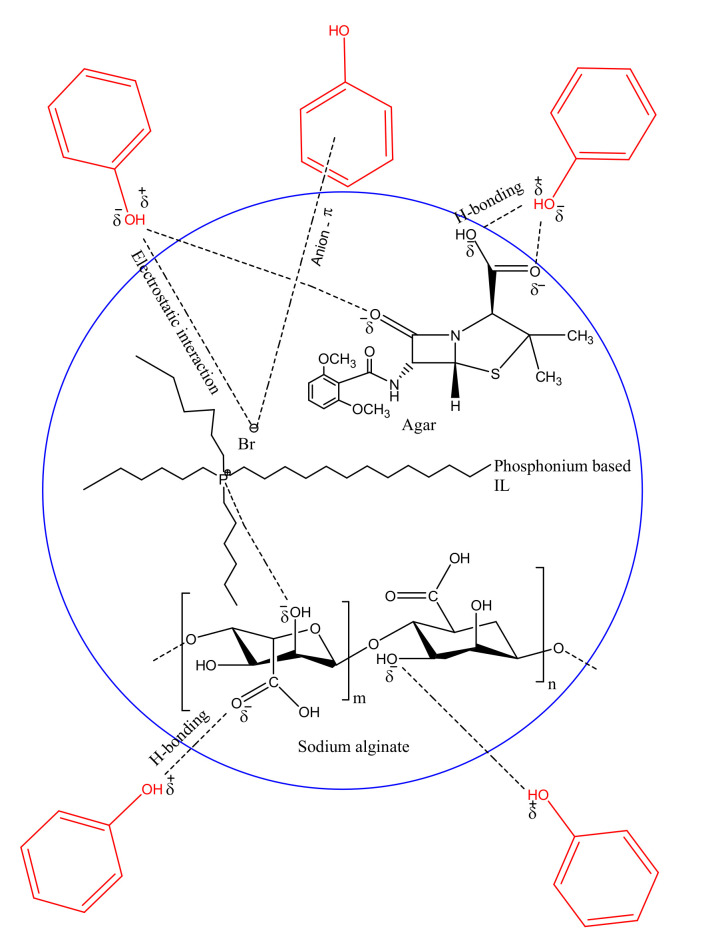
Proposed interaction forces between sodium alginate and IL and agar–IL–alginate beads with phenol.

Through experimental and analytical results, it is confirmed that the synthesized adsorbent has a good potential to treat PWW with low phenol concentrations. SEM images confirmed porous structure of the adsorbent. Therefore, one possible route for phenol adsorption can be through adsorptive pore-filling mechanism. Nguyen et al. reported dichlorobenzene, a phenol parental compound, and other higher molecular weight aromatics underwent same adsorptive mechanism in aqueous solution with a wood char biosorbent [51]. Results from parametric tests such as pH, contact time, initial phenol concentration, adsorption kinetics and models of isotherms are providing evidence to other probable adsorption mechanisms. For example, hydrogen-bonding adsorption mechanism and π-π stacking may be responsible for chemical species electrostatic interactions among each other. Evidence of the hydrogen-bonding adsorption mechanism of rutin–polyphenolic compound on adsorbents having amino, hydroxyl and carboxyl functional group is reported by Ye et al. [52]. Increasing the pH of the aqueous adsorbate solution results in a decrease in adsorption that indicated a substantial EI involvement between phenolate anions (C_6_H_5_O^−^) and IL’s phosphonium ion (PR_4_^+^) [52]. Sodium alginate has several active sites for hydrogen bonding, while C_6_H_5_O^−^ ions have a strong hydrophilic nature; hence, the existence of hydrogen bonds between phenol and hydrogen of –OH group present in alginate in the IL–agar–alginate beads during the adsorption process is inevitable. Oxygen of ketone groups present in agar also interacts with –OH group of agar. Furthermore, ion-induced dipole interaction exists between Br^−^ ion and the π-electrons present in phenol ring. In addition, the aromatic ring of phenol may also interact with the electron pairs available on the adsorbent surface to enhance the adsorption process. Paiman et al. highlighted the importance of adsorbent functional group interaction with aromatic adsorbate and thus maximizing the adsorption an adsorbent surface [53]. In summary, the factors that played a role in the successful adsorption process were pore filling, electrostatic forces, hydrogen bonding and π-π bonds. Figure 17 illustrates the proposed interaction forces between sodium alginate and IL and IL–agar–alginate beads with phenol.

## 4. Conclusions

In this work, IL–agar–alginate beads were successfully synthesized to effectively remove phenol from aqueous phase. The IL–agar–alginate beads were characterized by various instrumental techniques such as FTIR, TGA, SEM, EDX and PZC. EDX and FTIR results confirmed the successful incorporation of the IL into the IL–agar–alginate beads. Batch adsorption studies were carried out at 25 °C to investigate the effect of various parameters on the adsorption of phenol on the surface of the synthesized IL–agar–alginate beads. The maximum adsorption capacity of phenol on the IL–agar–alginate beads was achieved at pH 2 (25 °C) in 2 h. Furthermore, results of experimental data showed that the adsorption of phenol followed both pseudo-second order and Freundlich isotherm models. The optimum temperature was 25 °C for phenol adsorption onto the IL–agar–alginate beads. The thermodynamic results revealed that the adsorption of phenol is exothermic in nature and feasible and spontaneous at low temperatures. Moreover, the IL–agar–alginate beads were easily recycled and used again for the removal of phenol, in four sequential cycles, without a considerable loss in the adsorption efficiency. Based on the overall results, it can be concluded that IL–agar–alginate beads represent an efficient adsorbent for the removal of phenol from aqueous phase.

## Figures and Tables

**Table 1:** EDX data obtained from elemental analysis of adsorbent beads.

Element	Apparent Concentration	wt.%
C	0.55	56.83 ± 1.74
O	0.37	25.07 ± 1.65
P	0.15	2.59 ± 0.25
Cl	0.28	7.01 ± 0.36
Ca	0.31	7.68 ± 0.40
Br	0.03	0.84 ± 0.28
Total		100

**Table 2:** Kinetic parameters for the adsorption of phenol onto the IL–agar–alginate beads at 25 °C.

Kinetic Models	Parameters	50 mg/L	100 mg/L	150 mg/L
Pseudo-first order	*q_e_* (mg/g)	17.6	31.5	32.8
	*k*_1_ (min^−1^)	0.028	0.029	0.034
	*R* ^2^	0.913	0.990	0.960
Pseudo-second order	*q_e_* (mg/g)	23.5	45.4	53.8
	*k*_2_ (g/mg min^−1^)	0.002	0.002	0.002
	*R* ^2^	0.996	0.998	0.998

**Table 3:** Isotherm model parameters for the adsorption of phenol onto the beads at 25 °C.

Isotherm Model	Parameters	At 25 °C
Langmuir model	*q_m_* (mg/g)	159
	K_L_ (L/mg)	0.005
	R_L_	0.432
	*R* ^2^	0.835
Freundlich model	*K_f_* (mg/g)(L/mg)^1/n^	2.17
	N	1.46
	*R* ^2^	0.956

**Table 4:** Thermodynamics parameters of adsorption for phenol removal by IL–agar–alginate beads.

*C_i_* (mg/L)	*H*° (kJ mol^−1^)	*S*° (J mol^−1^ K^−1^)	*G*° (kJ mol^−1^)		
			298 K	308 K	318 K
150	−23.5	−78.8	−0.023	0.765	1.555

**Table 5:** Regeneration of phenol loaded IL–agar–alginate beads by hot water and hot methanol both at a temperature of 50 °C.

Cycle	Hot Water		Methanol	
	*q* (mg/g)	%*R*	*q* (mg/g)	%*R*
1	50.85	33.9	37.17	24.78
2	45.62	30.41	33.79	22.53
3	45.34	30.25	30.55	20.37
4	45.18	30.12	24.62	16.42

**Table 6:** Comparison of the adsorption capacity, pH and temperature of IL–agar–alginate beads for phenol removal with previously reported adsorbents.

Adsorbent	*q_e_* (mg/g)	pH	Temp. (°C)	Ref.
Borassus flabellifer fruit husk activated carbon (Pyrolysis)	13.97	2	-	[39]
Borassus flabellifer fruit husk activated carbon (H_2_SO_4_ activation)	13.42	4	-	[39]
Microorganism P. putida and acid-modified CESEP/ZIF-8	5.96	-	-	[40]
Rice husk	7.89	9	35	[41]
Acid-modified bentonite	6.8	4	20–25	[42]
Organo-functional groups	45.26	7–10	-	[43]
Tea waste biomass	9.49	5–9	-	[44]
Zeolite	4.31	3.3–8.6	40	[45]
Solvent extraction	3.45	7.5–8.5	40–60	[46]
Magnetic iron oxide nanopowder (MNM)	2.536	-	-	[47]
Methyl acrylic acid (MAA)-coated iron oxide nanoparticles (NPs)	950	7	-	[48]
Activated carbon alginate beads impregnated with IL	78.8	2	-	[49]
α-Fe_2_O_3_	16.17	2–6	60	[50]
IL–agar–alginate beads (*C_i_* = 150 mg/L)	16.28	2	25	This work

**Table 7:** Comparison of the removal efficiency and kinetics of IL–agar–alginate beads for phenol removal with previously reported adsorbents.

Adsorbent	Kinetics Parameters			(%) *R*	Ref.
	*k*	*q_e_*	*R* ^2^		
Borassus flabellifer fruit husk activated carbon (Pyrolysis)	0.022	4.06	0.99	83	[39]
Borassus flabellifer fruit husk activated carbon (H_2_SO_4_ activation)	0.024	4.38	0.99	80	[39]
Microorganism P. putida and acid-modified CESEP/ZIF-8	0.002	5.82	0.99	77	[40]
Rice husk	0.063	2.42	0.99	95	[41]
Acid-modified bentonite	0.05	4.58	0.99	94.2	[42]
Organo-functional groups	0.000	47.11	0.99	95	[43]
Tea waste biomass	0.004	9.64	0.89	82	[44]
Zeolite	0.02	13.87	0.99	-	[45]
Magnetic iron oxide nanopowder (MNM)	0.002	2.94	0.98	-	[47]
Methyl acrylic acid (MAA)-coated iron oxide nanoparticles (NPs)	-	1	0.99	90	[48]
Activated carbon alginate beads impregnated with IL	0.000	53.07	0.99	-	[49]
α-Fe_2_O_3_	0.23	10.66	0.99	-	[50]
IL–agar–alginate beads (*C_i_* = 150 mg/L, beads dosage = 1 mg/mL)	0.002	53.8	0.99	65	This work

## Data Availability

Not applicable.
